# From exposure to embedding: how interconnected social determinants drive health inequalities—a complex adaptive system dynamics approach

**DOI:** 10.3389/fpubh.2026.1923212

**Published:** 2026-09-08

**Authors:** Joachim P. Sturmberg, Carmel M. Martin, Stewart W. Mercer, Mark J. DeHaven, Holger Pfaff, Jeanette M. Bennett

**Affiliations:** 1 Faculty of Health and Medicine, School of Medicine and Public Health, University of Newcastle, Callaghan, VIC, Australia; 2 Department of Medicine, Nursing and Allied Health, Monash Health, Monash University, Clayton, VIC, Australia; 3 Department of Primary Care and Multimorbidity, Usher Institute, College of Medicine and Veterinary Medicine University of Edinburgh, Edinburgh, United Kingdom; 4 Academy for Research on Community Health, Engagement, and Services (ARCHES), University of North Carolina, Charlotte, NC, United States; 5 Faculty of Medicine and University Hospital Cologne, Institute for Medical Sociology, Health Services Research and Rehabilitation Science (IMVR), University of Cologne, Cologne, Germany; 6 Department of Psychological Science, StressWAVES Biobehavioral Research Lab, The University of North Carolina at Charlotte, Charlotte, NC, United States

**Keywords:** biological embedding, complex adaptive systems, health, health care system, health policy, psychoneuroimmunology, social determinants of health

## Abstract

The social determinants of health (SDOH)—including socioeconomic position, education, housing, and social networks—are fundamental drivers of health inequalities worldwide. These inequalities persist because social determinants interact dynamically across time and context, with health emerging from the interplay of social, political, environmental, and biological processes in which determinants act simultaneously as causes and consequences. Early-life exposures and historical conditions produce enduring physiological vulnerability through biological embedding, encompassing stress dysregulation, inflammation, and epigenetic modifications. Feedback loops, non-linearity, and path dependence stabilise disadvantage across the life course and across generations—mechanisms that explain why interventions targeting isolated risks or single factors often yield modest or context-dependent effects. A complex adaptive systems perspective reconceptualises health inequalities as emergent outcomes of these interacting social–biological processes and identifies leverage points where systemic, multi-level interventions can shift trajectories. Promising approaches include coordinated, place-based strategies, relational continuity in primary care, and strengthened social infrastructure.

## Introduction

*If you could at last change the world, would you step up and do it?*

Bertolt Brecht, *The Measures Taken,* 1930

The social determinants of health (SDOH) have long been recognised as fundamental drivers of health inequalities (1, 2). Across countries and historical periods, morbidity and mortality consistently follow steep social gradients (3) that are systematic, avoidable, and unjust. Yet despite decades of empirical documentation, health inequalities remain stubbornly persistent, and interventions targeting individual determinants have delivered only modest and uneven gains.

This persistence points to a deeper explanatory problem. The challenge is not a lack of evidence about what the social determinants are, but how they operate together. Much of the literature treats social determinants as separable exposures whose effects can be estimated independently and combined additively (1–5), obscuring a critical feature of social disadvantage—determinants interact, adapt, and accumulate over time in ways that fundamentally shape system behaviour.

Health inequalities do not arise from isolated risks but from the dynamic interplay between social, political, commercial, environmental, and biological processes. Determinants function simultaneously as causes and consequences (4), feedback loops that stabilise advantage for some and disadvantage for others. These interactions are non-linear, context-sensitive, and path dependent, with commercial actors increasingly deepening inequalities by structuring the environments in which people live, work, and consume.

Rudolf Virchow argued in 1848 that “*medicine is a social science and politics is nothing else but medicine on a large scale*” (6)—poor health is a political outcome, not a technical failure, shaped by power, social organisation, and material conditions. Contemporary evidence confirms that health inequalities reflect collective decisions about resource distribution and protection within societies.

Understanding health (7–9) and the SDOH requires a complex adaptive systems (CAS) perspective, shifting attention from individual determinants to system dynamics such as interdependence, feedback loops, adaptation, and biological embedding. The primary focus is on the interaction patterns through which social conditions become biologically consequential and self-reinforcing across the life course.

This article first outlines the political and resource-based foundations of social health inequalities (10, 11), then examines how social disadvantage translates into patterned biological vulnerability (12, 13). A CAS framework is introduced to explain why inequalities persist despite extensive intervention and to illustrate how systemic approaches at community, primary care, and individual levels can engage leverage points capable of altering system trajectories. The analysis concludes that lasting reductions in health inequalities require system-level interventions rather than those targeting isolated social risks.

## Defining the social determinants of health

**Figure 1:**
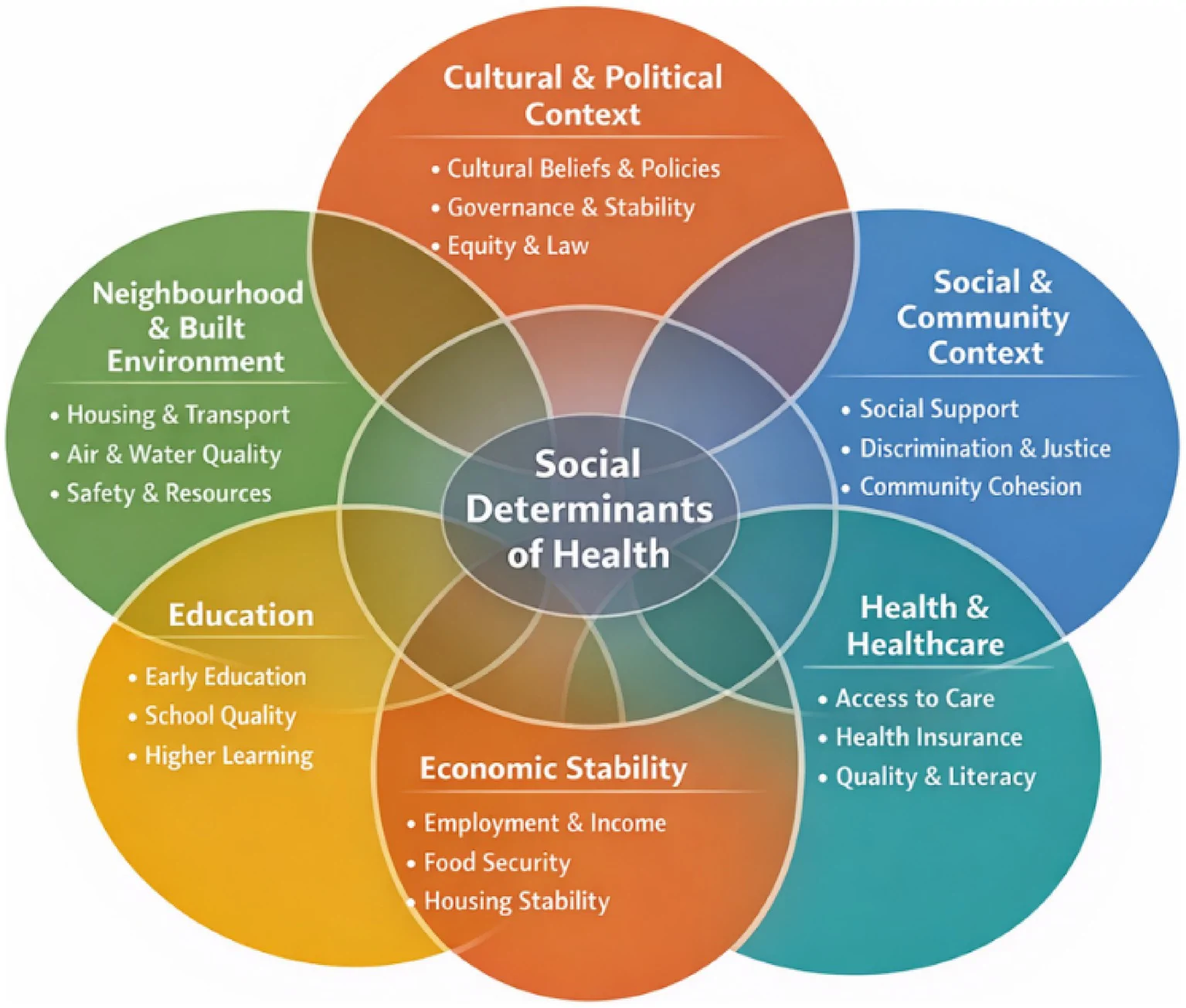
The overlapping features of the social determinants of health.

The SDOH encompass multiple overlapping domains (14)—cultural and political context, neighbourhood and built environment, social and community networks, education, economic stability, and healthcare access—that shape exposure to risk and protection across the life course and influence both immediate outcomes and longer-term health trajectories (Figure 1).

The core dynamics of poverty and marginalisation appear similar across levels of national income (11). While low-income countries face additional burdens from poor infrastructure, the disease burden attributable to social determinants shows striking similarities across low, middle, and high-income settings (11, 15). Even in high-income countries, where basic material needs are largely met, health inequalities remain pronounced and follow strong social gradients. In the United States, for example, the mortality burden from low educational attainment, income inequality, racial segregation, and weak social support rivals that of major behavioural and pathophysiological risk factors (16).

Taken together, social determinants are political in origin, profound in consequence, and inseparable in effect, operating across multiple levels (from the individual to the population) and timescales (from early-life exposures to later-life disease accumulation) (17–22). Yet despite well-established descriptive epidemiology, key questions remain—why do health inequalities persist across the life course, show marked context sensitivity, and resist interventions targeting single determinants?

What is missing is not more documentation of associations, but a framework that explains how social determinants interact over time to produce self-reinforcing patterns of health and disease—and why these patterns resist linear policy responses. Rather than treating social determinants as a list of discrete exposures, this interconnection calls for understanding them as elements of a dynamic system. The next section introduces a CAS perspective to clarify how interdependence, feedback loops, path dependence, non-linearity, and context sensitivity shape social determinant behaviour—providing the conceptual foundation for understanding how social disadvantage becomes entrenched and why effective responses must engage system dynamics rather than isolated factors.

## From social determinants to system dynamics—a complex adaptive systems perspective

Social disadvantage is not a set of discrete, independent risk factors but a patterned system of interlocking conditions that evolve over time. Health inequalities persist because the SDOH cluster, interact, and adapt, creating trajectories of accumulating vulnerability (17–22); yet conventional analytic approaches struggle to account for this, focusing on individual exposures rather than system behaviour as-a-whole (23, 24).

A CAS perspective offers a framework for understanding these dynamics. CAS are defined by interdependence, non-linearity, feedback loops, path dependence, and adaptation—properties that map closely onto the observed behaviour of the SDOH, where outcomes emerge from interactions among components rather than linear cause–and–effect relationships. The consequences of income, education, employment, housing, or social support cannot therefore be understood independently of each other or of the wider political and institutional context in which they are embedded (25–27).

Central to this account is the concept of emergence. Emergent properties are characteristics of a whole that arise from the organised interaction of its parts, are not possessed by those parts in isolation, and cannot be predicted by aggregating them; health (9), and by extension health inequality, is such a property.

This understanding is shared with critical realism, which treats social structures as real entities occupying a stratified reality, possessing causal powers that emerge from—but are not reducible to—the relations between their members (28, 29). The two traditions are complementary rather than competing (30). Critical realism supplies the ontological grounding: it treats emergent social structures as genuinely causal, countering the objection that systems language is merely metaphorical, and it brings a critical concern for power, inequality, and the conditions of social transformation. A complex adaptive systems perspective supplies the dynamic account of how those causal powers are exercised, reproduced, and occasionally overturned over time—through feedback, non-linearity, adaptation, and path dependence.

In short, CAS shows *how* emergent patterns unfold; critical realism explains *why* they carry causal and political significance. Both reject the reduction of population health patterns either to aggregated individual attributes or to structural determination alone, and both therefore preserve a role for agency within patterned constraint. On this account causation is itself reciprocal, with effects feeding back to reshape their own causes and system transformation remaining open to human agency rather than mechanically determined (31).

This shift helps address several persistent puzzles: the robustness of social gradients despite extensive adjustment for known risk factors; the pronounced context sensitivity of interventions, with similar programmes producing divergent outcomes across settings; and the limited long-term impact of policies targeting single determinants or behaviours. Rather than treating these as anomalies or implementation failures, a CAS lens recognises them as expected properties of complex socio–biological systems.

Framed this way, a complex adaptive systems account does not so much displace existing traditions in social epidemiology as specify what it adds to them. The theory of fundamental causes explains why socioeconomic gradients persist even as the specific diseases and risk factors change, because flexible resources are redeployed to whatever protects health at a given time (32). Life-course epidemiology documents how exposures accumulate and cluster along developmental trajectories (33); and eco-social theory describes how those exposures are embodied, becoming biologically expressed over time (34).

What a CAS lens contributes is an explicit account of the system behaviour these frameworks presuppose but do not formalise: the feedback loops through which resources and risks are reproduced, the non-linearity and tipping points that render gradients resistant to marginal change, and the path dependence and adaptation through which interventions are absorbed or deflected. Its distinctive claim is therefore not that social conditions matter—that is well established—but that their patterning is an emergent property of interacting dynamics, and that leverage lies in those dynamics rather than in any single determinant.

### Limitations of determinant-based models

Much of the SDOH literature treats social conditions as separable exposures whose effects can be estimated independently and added together. This approach has been methodologically productive, generating extensive evidence linking income, educational attainment, and neighbourhood deprivation to morbidity and mortality. However, it abstracts away from the processes that make disadvantage durable, including how social conditions co-occur, reinforce one another, and are reproduced through institutions and policies.

The empirical consequences are clear. Even after adjustment for biomedical and behavioural risk factors, substantial residual gradients in morbidity, mortality, and multimorbidity remain. Studies across multimorbidity (35, 36), cardiovascular disease (37, 38), and cancer (39) show that traditional risk factors explain only part of the outcome gap between more and less advantaged groups, with large, structured differences persisting across populations and disease domains (Table 1). These residuals are unlikely to be fully explained by unmeasured individual factors alone (36), suggesting that the underlying system, how resources, risks, and protections are patterned and reproduced, requires explanation.

**Table 1:** Examples from the literature of the effect of SDOH on health outcomes.

	Study focus	Outcome differences
Multimorbidity	People with >4 conditions and low educational level (assessed at the individual patient level) (35)	Total Mortality 6-times higher mortality than those with high 14% higher premature (< 75 yrs) mortality educational attainment Total number of diseases moderate mortality differences Lifestyle factors and QoL impact mortality at every educational level
	Biomedical and behavioural risk factor effects (smoking, alcohol consumption, diet, body mass index (BMI), physical activity) in relation to socioeconomic position (area-based deprivation and household income) (36)	Lifestyle and behaviour risk factors associated with the development of multimorbidity explain only 40.8% of the differences between socioeconomic conditions
Cardiovascular disease	SEP (education and a household wealth index) and risks for CVD (tobacco use, history of hypertension, diabetes, psychosocial factors, diet, physical activity, and physical measures) on health outcomes (composite of cardiovascular deaths, strokes, myocardial infarction, and heart failure), cardiovascular mortality, and all-cause mortality) between low, middle-income and high-income countries (38)	Low educational attainment is associated with a significant increased CFR across low and middle-income countries Wealth had minimal impact on outcomes in low and middle-income countries CVD risk factors were better in low and middle-income countries Health care is poorer in low and middle-income countries compared with high-income countries
	Low SEP and its association with premature cardiac events and mortality in the US (37)	Low SEP doubles the risk of MI and cardiac mortality Only 40% of event differences are explained by traditional risk factors (The ideal levels for traditional risk factors – BP, LDL-cholesterol, prior history of diabetes or CVD, diabetes, BMI, and smoking)
Cancer	Whole-of-population cancer-specific mortality data by socioeconomic position, as measured by education level in adults aged 40–79 (39)	32% of cancer deaths in men and 16% in women (but up to 46 and 24%, respectively in Baltic/Central/East-Europe) were associated with educational inequalities Nordic-countries, with a long-established tradition of equitable welfare and social justice policies, witness increases in cancer inequalities among women

BP, blood pressure; CFR, case fatality rate; CVD, cardiovascular disease; LDL cholesterol, low-density lipoprotein cholesterol; QoL, quality of life; SEP, socio-economic position.

Policy responses often mirror these analytic limitations. Interventions tend to target single determinants chosen for their measurability, administrative feasibility, or short-term impact, including lifestyle interventions, disease-specific programmes, and narrowly framed social policies. While these can improve proximal indicators, they rarely alter long-term inequality gradients because their effects are context-dependent or transient (40). This reflects a deeper limitation of reductionist approaches: if interventions do not change feedback loops or the ways determinants interact, they are less likely to shift system trajectories in sustained ways.

A CAS perspective addresses this limitation by shifting attention from isolated determinants to system behaviour, arising not from discrete risks but from the dynamic interplay of social, political, and biological processes over time. Its value lies not in terminology alone, but in its ability to organise empirical observations, reveal patterns that reductionist approaches miss, and identify leverage points capable of producing sustainable change.

### Interdependence and reciprocity

Interdependence is a defining feature of CAS. Applied to SDOH, this means the effect of any single determinant depends on its relationships with others and the broader environment. Low income illustrates this: its health effects vary with co-occurring housing, education, and welfare policies, just as poor housing outcomes depend on income security, social cohesion, and healthcare access. Changes in one domain thus reverberate across others, sometimes amplifying and sometimes dampening overall effects.

Reciprocity further complicates linear cause-and-effect notions. Poor health restricts educational attainment, employment stability, and income, reinforcing adverse social conditions and limiting access to resources (36). SDOH thus act simultaneously as causes and consequences (4) of health, eroding the distinction between “upstream” and “downstream.” This co-evolution appears, for example, in trajectories from early mental health problems to later unemployment, substance use, and cardiometabolic disease (41).

### Non-linearity and thresholds

Non-linearity, a core CAS feature, means inputs do not produce proportionate outputs. In health inequalities, small improvements in one SDOH may have a limited impact if other constraints persist, while changes at leverage points can yield disproportionate benefits. Modest income gains often fail amid housing insecurity and employment instability.

Non-linearity also implies thresholds and tipping points. Individuals and communities may absorb substantial adversity before adaptive capacity is overwhelmed, after which decline accelerates and resists reversal (14). Such thresholds reflect both social context and biological embedding, including allostatic load and declining resilience (14, 42). This has policy implications: interventions arriving late in the life course or targeting narrow domains may fail to shift entrenched trajectories despite improving specific indicators.

### Feedback loops and the stabilisation of inequality

Feedback loops provide insights into why health inequalities persist. Reinforcing loops occur when disadvantage in one domain amplifies further disadvantage or ill health, deepening initial vulnerabilities. Childhood adversity illustrates this: early socioeconomic hardship increases risks of educational underachievement and precarious employment, exposing individuals to chronic stress, material insecurity, and harmful environments (43). This contributes to mental health problems and chronic disease, which further limit employment, income, and social participation, closing the self-reinforcing cycle.

Longitudinal studies are consistent with this pattern, showing clustered disease onset and characteristic sequences among socioeconomically disadvantaged groups. Mental health conditions often emerge early, followed by substance use disorders, and are diagnosed early compared to physical health conditions like cardiometabolic disease, and other stress-related conditions (41), patterns not easily explained by models treating diseases as independent events, but consistent with system dynamics where chronic adversity, constrained agency, and biological wear drive clustering and progression.

However, when reinforcing loops dominate and protective structures—such as strong social networks, accessible primary care, or responsive social protection—weaken, inequalities become entrenched, and short-term interventions yield only temporary relief, unless tipping points—whether from macro-level disruption or grassroots social mobilisation—shift the system into a different regime.

### Path dependence, context sensitivity and adaptation

Path dependence means current system behaviour reflects prior states and historical events. Early-life conditions and past policy choices continue to shape health gradients: childhood socioeconomic disadvantage disrupts educational trajectories, social networks, and employment prospects, while becoming biologically embedded through altered stress regulation, immune function, and neurodevelopment. At the macro level, histories of segregation, welfare retrenchment, labour market deregulation, and colonial dispossession leave institutional and spatial legacies that pattern contemporary exposure and resilience (6, 22, 37, 43).

Path dependence helps explain why similar interventions yield different effects across contexts. Housing or healthcare access improvements may yield substantial gains when supportive social infrastructure is in place, but only modest benefits when other determinants remain highly adverse. This heterogeneity aligns with CAS expectations, as interventions act on systems with distinct histories, configurations, and feedback structures.

Adaptation, another core CAS property, means individuals, families, communities, and institutions respond to interventions in ways that may reinforce or undermine intended effects. Health system reforms illustrate this: expanded coverage paired with increased administrative complexity can exclude those least able to navigate bureaucracy. Individuals facing persistent insecurity may prioritise immediate needs over long-term health, a rational short-term strategy that devalues/ignores future risk (44, 45). These responses underscore the need for iterative, learning-focused policies sensitive to local conditions rather than fixed, one-size-fits-all solutions.

Taken together, CAS concepts recast health inequalities as emergent properties of interacting social, institutional, and biological processes. They explain why linear, determinant-specific interventions often fail while identifying leverage points frequently shift system trajectories. Understanding these leverage points requires examining not only how social conditions are organised but also how they become embodied.

## Biological embedding—how social disadvantage becomes physiologically entrenched

A CAS perspective explains how social conditions create persistent inequalities through biological embedding, progressively inscribed into bodies over time. Biological embedding describes processes by which social and environmental exposures become incorporated into physiological systems, shaping development, stress regulation, immune function, metabolism, and behaviour across the life course (35–37). Biology is not a passive recipient of social influences but an active, adaptive system responding to prevailing conditions in ways that can enhance short-term survival but increase long-term disease risk.

Multiple interacting mechanisms contribute to biological embedding. Early-life adversity, chronic psychosocial stress, material deprivation, and social marginalization alter neuroendocrine pathways, inflammatory responses, epigenetic profiles, and neural development (46–50), producing durable shifts in biological set points and adaptive capacity. In CAS terms, biological embedding represents internal path dependence—previous social experiences reshape the starting conditions for subsequent responses to stress, opportunity, and intervention.

### Sensitive periods and life-course trajectories

Life-course epidemiology shows that exposures during sensitive developmental periods—particularly in utero, early childhood, and adolescence—have disproportionate later health effects (44–46). Socioeconomic disadvantage in these phases alters stress responsivity, impairs cognitive and emotional development, and elevates risks of adult cardiometabolic, respiratory, and mental health conditions, even after accounting for adult circumstances.

Such findings explain why health inequalities emerge early and persist across decades and generations. Children from disadvantaged environments enter adulthood with higher allostatic load, reduced reserve capacity, and altered expectations of control and opportunity (43, 51–53), embodied differences that shape responses to future stressors and interventions, influencing both disease risk and the effectiveness of preventive or therapeutic efforts.

### Chronic stress and allostatic load

Chronic psychosocial stress centrally links social conditions to biological embedding. Repeated hypothalamic–pituitary–adrenal axis activation and parasympathetic withdrawal amid material insecurity, social exclusion, discrimination, or constrained agency dysregulate stress-response systems and accumulate allostatic load (43, 51–53). Allostatic load is a composite index of neuroendocrine, metabolic, cardiovascular, and inflammatory markers that accelerates biological ageing and immune dysfunction (21, 42, 54).

Exposure to chronic stress is not randomly distributed but strongly patterned by social position (SEP). Those in lower SEP face unstable employment, inadequate housing, unsafe environments, debt, and discrimination, with fewer resources to buffer these stressors. This reinforces the feedback loops through which social disadvantage and biological vulnerability co-evolve, contributing to earlier disease onset, more rapid progression, and higher mortality among disadvantaged groups (12, 13).

### Inflammation as a cross-cutting pathway

Inflammation provides a second key pathway through which social conditions become biologically embedded (55, 56).

From a systems perspective, inflammation acts as a cross-cutting mediator, connecting diverse social exposures to multiple downstream outcomes (55, 56). Once established, chronic low-grade inflammation can influence appetite, mood, energy regulation, and immune competence, affecting health behaviours, susceptibility to infection, and treatment response (55), creating additional pathways through which social conditions shape both disease risk and health system effectiveness.

### Epigenetic modification and intergenerational transmission

Epigenetic mechanisms illustrate how social conditions leave lasting molecular signatures without altering DNA sequences. Early-life adversity, chronic stress, and nutritional deprivation are linked to epigenetic changes affecting gene expression involved in stress regulation, immune function, and neurodevelopment (46–50), with some modifications persisting into adulthood and transmitting across generations through germline effects or social pathways such as parenting, neighbourhood context, and cultural practices. These findings raise the possibility that the consequences of social disadvantage span multiple lifetimes, although human evidence for germline transmission specifically remains limited and contested.

The epigenetic evidence for embedding is less settled than usually presented: social epigenetics in particular is limited by proxy (peripheral) tissues, patchy epigenome coverage, and small, predominantly cross-sectional samples from which directionality cannot be inferred (57). Associations between early-life exposure and specific methylation sites often fail to persist across development, with different loci implicated at different ages (58). The associations are also modest and inconsistent: a recent meta-analysis linked social disadvantage to accelerated epigenetic ageing, but the effects were often weak and were further attenuated by adjustment for smoking and body mass index, with the evidence skewed toward high-income countries (59). Robustness in this field is therefore better pursued through triangulation across designs, tissues, and populations with unrelated sources of bias than through replication alone (57, 60). Furthermore, recent advances highlight that focusing on epigenetic changes within one physiological system may underestimate biological ageing within the whole person and contribute to the inconsistent findings (61).

### Embedding, agency, and policy

Biological embedding does not imply biological determinism. Embedded physiological changes are best understood as adaptive responses to particular social environments rather than irrevocable damage (62, 63). In other words, epigenetic changes result from the individual’s interaction with their environment. Their maintenance is conditional on the persistence of the conditions that produced them. Where circumstances improve, physiological trajectories can shift: relational and psychosocial interventions alter inflammatory gene expression in adults raised in low-socioeconomic-position households and in families exposed to early adversity (62, 63), and epigenetic or biological ageing measures are increasingly used as modifiable outcomes in randomised evaluations of behavioural programmes and income transfers (59). Structure, agency, and biology are thus mutually constituting rather than sequentially determining—which is what an emergentist account implies, and what a deterministic reading of embedding would preclude.

Once established, however, these adaptations can constrain the effectiveness of later interventions, especially those introduced late in the life course or focused narrowly on behaviour change or disease-specific treatment. Efforts to promote healthy lifestyles often fail when they ignore the chronic stress, constrained agency, and material scarcity that shape both decision-making and physiological responsiveness.

Recognising biological embedding raises the ethical and political stakes of SDOH. When social disadvantage becomes embodied, health inequalities can no longer be framed as individual choices but reflect sustained exposure to adverse conditions shaped by policy and institutional arrangements.

**Figure 2:**
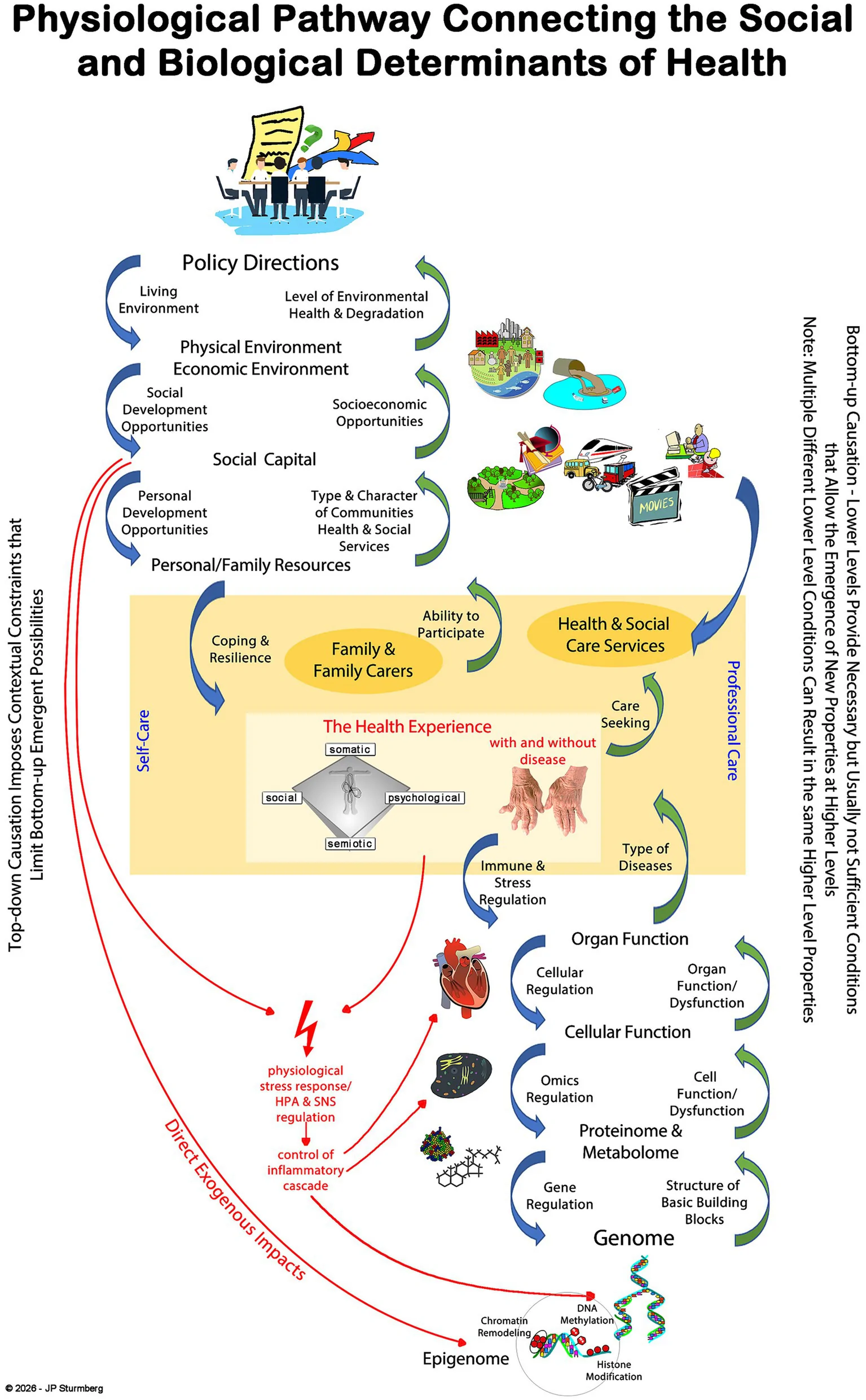
The CAS relationships of embedding. The vortex as a metaphor to link the external and biological domains of health.

Finally, biological embedding feeds back into the social system. Health impairments from embedded disadvantage limit educational attainment, employment, caregiving, and civic participation, reinforcing the social conditions that produced them. Embedding thus serves as both an outcome of system behaviour and a driver of future dynamics. This occurrence underscores the need for early life-course interventions that operate across multiple domains and intentionally reshape the socio-biological interface, rather than relying on downstream clinical care to compensate for upstream social harms (Figure 2).

Biological embedding mechanistically explains how social conditions produce durable health inequalities, but its implications extend beyond biology. If social disadvantage becomes biologically incorporated through repeated stress, material deprivation, and constrained agency, then policy and practice necessarily shape the upstream conditions to which bodies adapt. From a CAS perspective, policy acts as a system intervention by altering exposure patterns, feedback loops, and adaptive pathways over time, rather than merely providing services or mitigating risks. Effective SDOH responses thus target the socio-biological interface itself, influencing how vulnerability accumulates, resilience emerges, and health trajectories stabilise or shift across the life course.

## Policy and practice implications—addressing SDOH through a complex adaptive systems approach

Understanding the SDOH as interconnected, dynamic drivers of both biological and social outcomes requires rethinking how policy and practice are conceived. Health inequalities arise through the co-evolution of social conditions and biological responses, mediated by feedback loops, non-linearity, and adaptation. Policy and clinical practice are not separate tools acting on isolated determinants but integral components of the systems shaping how individuals and communities adapt over time.

This reframing shifts attention to the upstream conditions structuring adaptive responses. Social policies (64) and clinical practice influence the same underlying dynamics—how social adversity becomes biologically embedded and manifests as health or disease. Several CAS-derived principles become actionable:

- *Optimise interdependencies*: Coordinated action across housing, education, employment, and health generates synergistic effects unavailable from siloed interventions.
- *Engage multiple levels*: Interventions must span individuals, communities, organisations, and policy structures for sustainability.
- *Work with feedback*: Reduce reinforcing loops of disadvantage while strengthening balancing loops of resilience.
- *Enable adaptation*: Context-sensitive implementation allows local evolution rather than uniform solutions.
- *Target leverage points*: Modest changes at high-influence nodes (social cohesion, income security, relational continuity) can shift system trajectories.

The following examples illustrate CAS principles across system levels. These are deliberately not blueprints as effects depend on the history, configuration, and feedback structure of the receiving system. What worked in Dallas or in Scotland’s most deprived practices cannot be lifted intact into another setting. What transfers is the process, not the intervention—convening those with local knowledge, identifying the constraints that actually bind, and adjusting as the system responds. The deliverable is a disciplined way of working, not a specification of what to do.

These are illustrations, not proof of the mechanisms: apart from trials such as CARE Plus, they were not designed to measure feedback, adaptation, or path dependence directly. Doing so is hard—the system’s openness confounds attribution, effects outlast most evaluation cycles, and conventional trials assume the stability complexity denies. The methods suited to it—systems mapping, network analysis, agent-based modelling, realist evaluation—remain unevenly applied, and most complex-systems evaluations capture one moment rather than tracking change.

### Community partnerships

The Dallas community health science partnership aligned the actions and assets of health care providers and community-based organisations in the Southern Sector—a predominantly African American area of concentrated poverty and severely constrained healthcare access—built on the recognition that health cannot improve unless social conditions are addressed through the community’s own structures (65, 66), embodying the CAS principle of optimising interdependencies.

Project Access Dallas (PAD) targeted the most immediate barriers: uninsured residents’ inability to access and navigate a healthcare system not designed for them. It deployed community health workers (CHWs) recruited from within the community (65, 66) who facilitated appointments with volunteer providers, provided emotional support, and linked clinical services with social needs. Rather than reforming the healthcare system wholesale, CHWs enhanced system connectivity by including structurally excluded people—a modest input producing a disproportionate benefit, consistent with CAS nonlinearity. PAD provided the impetus for the Baylor Scott and White Health and Wellness Centre, which embedded primary care and prevention within a city recreational facility, with participants showing a 21.4% reduction in emergency department visits and 36.7% reduction in inpatient care, resulting in cost reductions of 34.5 and 54.4%, respectively (66).

The GoodNEWS programme complemented this approach by leveraging existing community infrastructure (67). Local African-American church congregations—with established networks of trust, shared identity, and collective agency—represented pre-existing balancing feedback loops that the partnership could strengthen rather than create anew. Recruited as community partners, congregation-based lay health promoters delivered locally adapted wellness programmes addressing physical activity, nutrition, and psychosocial wellbeing. The church-based cohort showed better awareness, treatment, and control of hypertension, diabetes, and hyperlipidaemia than matched Dallas County African American populations—despite concentrated socioeconomic disadvantage—indicating that working with existing community infrastructure can shift biological risk trajectories rather than merely improving service access (67).

### Strengthening primary care—the deep end approach

Primary care serving the most disadvantaged populations exemplifies the *inverse care law*—where need is greatest, services are weakest (68). The Deep End project, led by GPs serving Scotland’s 100 most deprived practice populations, framed this as a CAS problem: not only under-resourcing but also misconfiguration, with capacity allocated inversely to need (69). Practitioners documented both the scale and nature of unmet need: patients presenting with clustered biological, psychological, and social complexity poorly suited to disease-specific protocols. Research confirmed that multimorbid patients in deprived areas receive no longer consultations than others, unlike in affluent areas, creating a systematic mismatch between complexity and care capacity (70). Deep End practices thus encounter biological embedding daily: stress dysregulation, inflammation, and cumulative adversity manifesting as multimorbidity, mental ill-health, and shortened lives.

The Deep End response applies the CAS principle of enabling adaptation: rather than imposing uniform protocols, it fostered locally appropriate, trust-based care through relational continuity, extended consultations, integrated teams including community link workers and welfare advisors, and practice collaboration (71). Universal proportionalism, i.e., funding proportional to socioeconomic need, served as the leverage point, realigning resources with complexity rather than headcount and reconfiguring the inverse care law. Evidence supports this at multiple levels: GP empathy in deprived areas is associated with better patient wellbeing, and extended consultations with greater patient enablement, indicating that relational quality is itself health-producing (70). The Links Worker Programme, embedding community links practitioners in seven Glasgow practices, showed that among patients with three or more contacts (45% of referrals), quality of life, depression, anxiety, and exercise improved significantly, with strong links to community resource uptake (69). This CAS pattern reveals outcomes hinge less on interventions than on relational engagement depth, operating through feedback activation rather than direct causation.

### Patient journey–focused care

High-utilising patients with complex social adversity reveal a core limitation of disease-centred care: standard models treat chronicity as discrete conditions rather than dynamic trajectories through phases of stability, instability, and crisis shaped by illness, social support, environment, and coping (72). This exposes the CAS problem of non-linearity: the same patient requires qualitatively different responses based on their trajectory position, not merely their diagnoses. Superutiliser programmes targeting utilisation patterns without consistently addressing underlying system dynamics consistently show minimal impact (73), as they modify surface signals while leaving generating feedback loops intact.

The Complex Adaptive Chronic Care (CACC) framework, developed by Martin and colleagues, applies CAS principles directly to individual care (74). Instead of diagnosis-centred organisation, CACC identifies the patient’s journey phase—stable, unstable, or chaotic—and tailors responses accordingly. Across two prospective cohorts, two-thirds of patients followed stable-complex journeys needing minimal intervention, one-quarter were unstable, and 7% required intensive chaotic-phase support (45). Chaotic patients required, on average, four times as many interventions as stable ones, a non-linear relationship missed by disease-specific metrics. The Patient Journey Record System (PaJR) implemented CACC via trained lay care guides who conducted regular conversational calls to monitor biopsychosocial trajectories in real time, with alerts reducing hospitalisations by 500 person-months (75). This approach was replicated in multicultural inner-city Melbourne (MonashWatch), demonstrating CAS adaptation across contexts. Throughout, the CAS principle of enabling individual-journey adaptation prevails: the system responds to whole-person perturbations rather than applying protocols, attenuating social adversity’s biological effects at the person-system interface despite unresolved upstream conditions.

Table 2 summarises the key CAS messages—these approaches succeed by working with system dynamics, reshaping interactions at the social-biological interface rather than targeting isolated risks. CAS thinking reveals leverage points where relatively modest investments (procedural equity, proportional funding, and relational continuity) can generate disproportionate system-wide benefits.

**Table 2:** Mapping illustrative examples to core CAS principles.

CAS Principle	Community partnerships	Strengthening primary care (deep end)	Patient journey–focused approaches
Interdependence	Links social support, health literacy, service navigation, and prevention, recognising that gains in one domain reinforce others	Integrates clinical care with social services, welfare advice, and community resources	Addresses biological, psychological, and social factors simultaneously rather than treating utilisation or disease in isolation
Multiple system levels	Operates across individuals, communities, organisations, and local policy contexts	Aligns individual care, practice organisation, funding models, and system leadership	Connects individual experiences with service design, digital tools, and organisational flexibility
Feedback loops	Strengthens positive feedback between social cohesion, healthier behaviours, and reduced acute care demand	Continuity and trust reduce crisis-driven use, reinforcing stability and engagement	Stabilised journeys reduce crises, which in turn reduce system strain and future vulnerability
Adaptation and context sensitivity	Place-based partnerships evolve according to local assets, histories, and trust	Locally adapted practice models emerge within enabling funding and policy conditions	Care models adjust dynamically to changing patient circumstances and life events
Leverage points	Procedural equity and trust act as high-leverage conditions for sustained change	Funding linked to need (universal proportionalism) reshapes system behaviour	Relational continuity and flexibility modulate stress and adaptive capacity
Emergence of the solution itself	The response was developed with the Southern Sector rather than for it; community health workers were recruited from within the community and priorities followed residents’ own account of what obstructed care	Practitioners in the most deprived practices set the agenda themselves; proposals emerged from collective deliberation among peers rather than from external specification	Responses are generated case by case from the patient’s own account of their journey rather than from a predetermined protocol

These accounts should not imply that systems approaches are easy to sustain. Each depended on conditions that are neither automatic nor durable: intersectoral governance arrangements surviving electoral and administrative turnover; funding horizons longer than the two to three-year cycles typical of programme grants; a workforce with the continuity and protected time to build relationships; and data-sharing across organisational boundaries. Where these were absent, comparable initiatives have stalled or regressed, and the inverse care law (68) describes precisely the tendency of resources to drain away from the settings where such work is most needed. The published record is also asymmetric, favouring initiatives that persisted long enough to be written up; failures and mixed results are correspondingly under-represented in the evidence drawn on here, and in the field more broadly.

## Conclusion

Health inequalities persist globally because SDOH operate as an interconnected, dynamic system rather than isolated risk factors. Health emerges from interacting social, political, commercial, environmental, and biological processes, with determinants serving as both causes and consequences of sustaining conditions. Early-life exposures and historical factors create lasting physiological vulnerability through biological embedding, in which chronic stress, inflammation, epigenetic changes, and neurodevelopmental adaptations convert social disadvantage into a persistent health risk that is reproduced across generations largely through social pathways. Feedback loops, non-linearity, and path dependence reinforce inequalities across life courses and generations, explaining why single-determinant interventions yield limited, context-dependent effects.

Effective responses demand systemic approaches that optimise interdependencies across domains, engage multiple system levels, and target high-leverage points capable of shifting trajectories. Among the most consequential yet under addressed interdependencies are commercial determinants of health: private-sector strategies shaping food environments, housing markets, labour conditions, and the political economy of welfare. These forces actively reproduce the systemic conditions that sustain disadvantage, making their address a precondition for the sustained effectiveness of other interventions. Community partnerships, Deep End primary care, and patient journey-focused approaches demonstrate that such leverage points exist and prove accessible through coordinated, relational, adaptive practice.

As one Deep End GP observed: “*People say that is just anecdote, it is not really evidence, but that’s nonsense. This is evidence. What these people tell us is evidence of whether the system is working or not. Whether society is functioning or not functioning*” (67). By recognising health as an emergent property of complex socio–biological systems, policymakers and clinicians can build enabling structures and coordinate action across levels to reshape feedback loops, reduce embedded vulnerability, and achieve sustainable change that transforms system trajectories rather than merely addressing isolated risks. Virchow’s insight, that politics is medicine at a large scale, has never been more empirically grounded. The question is no longer whether we understand the system, but whether we have the political will to act on that understanding, at its required scale and across its critical domains.

## Data Availability

The original contributions presented in the study are included in the article/supplementary material, further inquiries can be directed to the corresponding author.
